# Can classical statistics and deep learning converge on explainable, causally driven target discovery?

**DOI:** 10.1093/dnares/dsaf024

**Published:** 2025-09-15

**Authors:** Liyin Chen

**Affiliations:** Department of Ophthalmology, Massachusetts Eye and Ear, Harvard Medical School, Boston, MA 02420, United States

**Keywords:** causal representational learning, multi-omics integration, GWAS, deep learning, genomics

## Abstract

Understanding the molecular causes of complex diseases remains one of the most pressing challenges in biomedicine. Despite large-scale genome-wide association studies mapping thousands of risk loci, identifying which genetic variants truly drive disease remains difficult. Traditional statistical genetics has laid a strong foundation for variant discovery, but it often struggles to capture nonlinear interactions and cannot fully integrate the breadth of the interconnected multi-omics data. In recent years, deep learning approaches have shown promise in bridging these gaps: modelling high-order genetic interactions, uncovering latent biological structure, and enabling multi-layered data integration. However, most current deep learning models for genomics remain exploratory in nature, and issues such as susceptibility to overfitting, difficulties in interpretability, and the general lack of standardized evaluation frameworks have limited their widespread adoption for genomics research. In this review, we explore how traditional statistical and deep learning methods can be applied to uncover causal mechanisms in complex disease. We critically compare these two frameworks for their advantages and limitations in detecting genetic associations and prioritizing causal associations. Towards the end, we propose a future direction centred around hybrid models that blend the scalability of deep learning with the inferential power of statistical genetics. Our goal is to guide researchers in developing next-generation computational tools to uncover the molecular basis of complex diseases and accelerate the translation of genetic findings into effective treatments.

## 1. Introduction

Heredity is complex and most diseases such as Alzheimer’s Disease and diabetes are heterogeneous conditions whose phenotypes are influenced by both genetic and environmental factors. Along with the rise in population, these conditions are increasing and, therefore, putting a growing burden on the healthcare systems.^1^ Although the research on these complex multifactorial diseases has been going on for several decades, the developed treatments are mainly aimed at alleviating the symptoms rather than curing or preventing the disease.^2,3^ For many patients, this means years of trial-and-error treatment and little hope for true disease modification. This therapeutic gap shows an important need for therapeutic strategies which tackle the underlying causes of these conditions.

Target identification is the first and most important step of systematic drug discovery pipelines, as selecting the right molecular targets significantly increases the likelihood of developing a successful therapeutic agent. It has been showed that therapeutics that are backed by solid genetic evidence are much more likely to succeed in clinical trials as compared to those without genetic evidence.^4,5^ Specifically, drugs targeting genes with well-established genetic associations are more likely to succeed in Phases II and III of the drug development process, with genetic validation increasing the chances of drug approval by more than two times.

Thanks to the large sample sizes in population-scale datasets and advances in genome-wide association studies (GWASs), we now have a growing catalog of genetic loci associated with complex diseases. However, not all disease-associated genetic elements are causal. Many simply tag broader regions through linkage disequilibrium (LD), while others may lie in noncoding areas that influence gene expression in subtle, context-dependent ways. Consequently, without clear knowledge of the causal biology, therapeutics may address the symptoms rather than the underlying disease mechanisms. For example, BACE1 inhibitors for Alzheimer’s disease, designed to lower amyloid-beta levels, ultimately failed in late-stage trials due to off-target effects on synaptic proteins that lead to unexpected cognitive decline and severe adverse effects.^6^ This demonstrates the need for approaches that accurately identify causal architecture to inform the development of mechanism-based therapies for neurodegenerative diseases.

To identify causal genetic factors and inform drug development, statistical genetics has evolved beyond GWAS. A suite of post-GWAS methods have been developed to refine the list of loci identified from GWAS to pinpoint those that have putative causal relationship to the disease of interest in a functional context. For example, statistical fine-mapping methods narrow down causal variants within associated loci by disentangling true signals from LD; colocalization analyses integrate GWAS results with expression quantitative trait loci (eQTL) data to identify shared genetic drivers of both disease and molecular traits; Mendelian randomization (MR) leverages genetic variants as instrumental variables to infer causal relationships between modifiable exposures, such as gene expression or protein levels, and disease outcomes.^7^ These approaches collectively provide a statistical framework for prioritizing therapeutic targets with a strong mechanistic basis.

Although we have made strides in identifying genetic markers linked to diseases, traditional genetics approaches have inherent limitations in fully capturing the genetic complexity of complex polygenic diseases. The standard GWAS framework looks at how individual variants relate to disease risk and assumes these variants work independently. While this simplifies computation, it overlooks the nonlinear interactions among genetic loci that impact disease susceptibility. Earlier interaction models that try to detect significant interacting variants suffer from limited statistical power and computational inefficiency. The huge number of possible interactions among single-nucleotide polymorphisms (SNPs) grows exponentially with the number of variants, making exhaustive genome-wide interaction testing impractical. Furthermore, conventional post-GWAS approaches often model the effects between omics layers in a pairwise or sequential manner,^8,9^ overlooking the interdependent nature of molecular regulation where a variant's functional impact is conditional upon the state of other layers, such as the local epigenetic landscape. In diseases where genetic risk is influenced by highly regulated molecular pathways, this approach may miss important disease-driving mechanisms. For example, the impact of a variation on gene expression might vary depending on the situation influenced by factors, like chromatin accessibility, histone modifications or metabolic conditions.^10,11^ This kind of context-dependent regulation can only be readily understood by treating the multi-omics data as interconnected parts of a larger system and studying the causal relationships that shape disease development^12–14^

Deep learning approaches provide a new solution to tackle these challenges. They can learn nonlinear patterns in genomic data without requiring explicit interaction terms. Similarly, they excel in integrating high-dimensional multi-omics datasets. Unlike statistical frameworks that require predefined hypotheses about how omics layers interact, deep learning models can extract latent representations from each omics modality and identify cross-modal dependencies. Their capacity to integrate spatial and temporal omics data is especially useful for complex diseases, as many diseases evolve over time and differ across tissue regions, such as neurodegenerative disorders.^15^ One approach involves using autoencoders to condense diverse omics characteristics into embeddings that reflect variations underneath it all. Moreover, attention-based models and transformer designs take this a step further by assigning varying weights to features and effectively highlighting the most significant molecular cues, for a specific disease. By leveraging these approaches, deep learning makes it possible to construct comprehensive models of disease mechanisms that incorporate genetic, transcriptomic, epigenomic, and proteomic interactions in a unified structure.

While the potential of deep learning is considerable, it is important to acknowledge that its application to causal inference in genomics is still a rapidly evolving field. Many deep learning models are currently exploratory as compared to the traditional statistical methods, and there is an ongoing need to develop standardized evaluation frameworks, rigourous methods for assessing reproducibility, and quantifiable statistical confidence in their outputs. An important obstacle faced is the problem of overfitting where the model ends up capturing patterns to the training data instead of more general relationships relevant across different datasets. Another limitation is its lack of statistical rigour and interpretability. Traditional statistical genetics methods are built on well-established principles of hypothesis testing and causal inference. They provide quantifiable measures of uncertainty, such as *P*-values and confidence intervals, which are essential for evaluating the statistical significance and precision of findings. In contrast, deep learning models, in their naive form, often function as “black boxes,” making it difficult to understand the biological basis of their outputs. This lack of transparency hampers their effectiveness in validating causal relationships between genetic variants and disease phenotypes.

Combining the scalability of deep learning with the rigour of statistical genetics presents a promising path forward for uncovering causal mechanisms in complex diseases. Deep learning-based causal representation learning can model complex genetic interactions and integrate multi-omics data in a biologically meaningful way. These advancements enable comprehensive genome-wide discovery of putative causal genetic variants that drive disease processes. Integrating statistical principles of causal inference into deep learning-driven causal discovery can further ensure that these computational discoveries translate into robust biological insights. This integration not only mitigates overfitting of the data but also refines and enhances the statistical confidence of the identified causal relationships.

In this review, we explore the evolving landscape of computational approaches to causal inference in complex disease. We begin by reviewing traditional statistical approaches, including GWAS and post-GWAS analyses, highlighting their contributions and limitations in target discovery. We then examine how deep learning is reshaping the field, with a focus on epistasis modelling and multi-omics integration. We critically assess two main limitations of these deep learning methodologies: overfitting and interpretability. Finally, we outline future research directions focused on developing integrative frameworks that merge deep learning’s ability to discover novel genetic drivers with statistical methodologies that ensure robust causal interpretation. Our goal is to guide researchers in field to navigate next-generation computational tools for uncovering the molecular basis of complex polygenic diseases.

### 1.1. Established statistical genetics approaches

GWAS leverages high-throughput genotyping technologies and statistical models to analyse millions of SNPs across the genome, aiming to uncover genotype–phenotype associations. The predominant statistical frameworks in GWAS include fixed-effect and linear mixed-effect models, each tailored to specific study designs and population structures. The fixed-effect model assumes a consistent effect of a genetic variant across all individuals in the study and is particularly suited for homogeneous populations with minimal population structure.^16^ It is computationally efficient and straightforward, making it ideal for initial scans of well-matched cohorts. However, they do not inherently address confounding factors such as relatedness or population structure. To mitigate these issues, researchers commonly calculate identity-by-descent metrics to exclude closely related individuals and adjust for population stratification by including principal components derived from genetic data as covariates in association analyses. Despite these adjustments, fixed-effect models may still fail to fully capture complex confounding effects, potentially leading to residual biases in highly diverse populations. The linear mixed-effect model provides an advantage by incorporating a random effect term in addition to the standard fixed-effect covariates like principal components. This random effect uses a genetic relationship matrix to account for confounding from the full spectrum of genetic relatedness, including both cryptic relatedness and the subtle population substructure not fully captured by top principal components.^7^ By modelling these confounding factors, it reduces the risk of false positives, especially in diverse populations. While computationally more demanding than fixed-effect models, it is particularly advantageous in large-scale studies or meta-analyses involving heterogeneous datasets.

Large-scale biobanks have been instrumental in advancing GWAS by providing extensive genetic and phenotypic data. The UK Biobank (∼500,000 genotyped participants with imaging, biomarker and linked-EHR data),^17^ BioBank Japan (∼260,000 disease-ascertained participants),^18^ the NIH All of Us Research Programme (>500,000 participants of whom 77% are from historically under-represented groups),^19^ and FinnGen (nation-wide Finnish network linking registry phenotypes for >400,000 people)^20^ are some of the major biobanks. Complementary to these biobanks are disease-focused consortia, such as the International Glaucoma Genetics Consortium (IGGC)^21^ and the Global Lipids Genetics Consortium (GLGC).^22^ IGGC meta-analyses 34,179 primary open-angle glaucoma cases and 349,321 controls across >20 cohorts, identifying 127 risk loci consistent across ancestries. GLGC pools genome-wide data from ∼1.65 million individuals of multiple ancestries to map hundreds of loci influencing LDL-C, HDL-C, triglycerides and total cholesterol. These consortia harmonize data from many cohorts and public biobanks and release high-powered, trait-specific summary statistics that complement individual-level biobank resources.

While GWAS have provided unprecedented insights into the genetic architecture of complex diseases, translating these associations into actionable drug targets requires post-GWAS methodologies to identify the biological mechanisms that cause the disease of interest.^23^ The challenge lies in resolving the causal variants and genes from the statistical associations observed in GWAS, as most significant loci encompass multiple variants in LD and often reside in noncoding regions. To address these challenges, a plethora of post-GWAS methods have been developed, which leverage fine-mapping and omics data to prioritize causal variants and genes.^24^

**Fig. 1.**
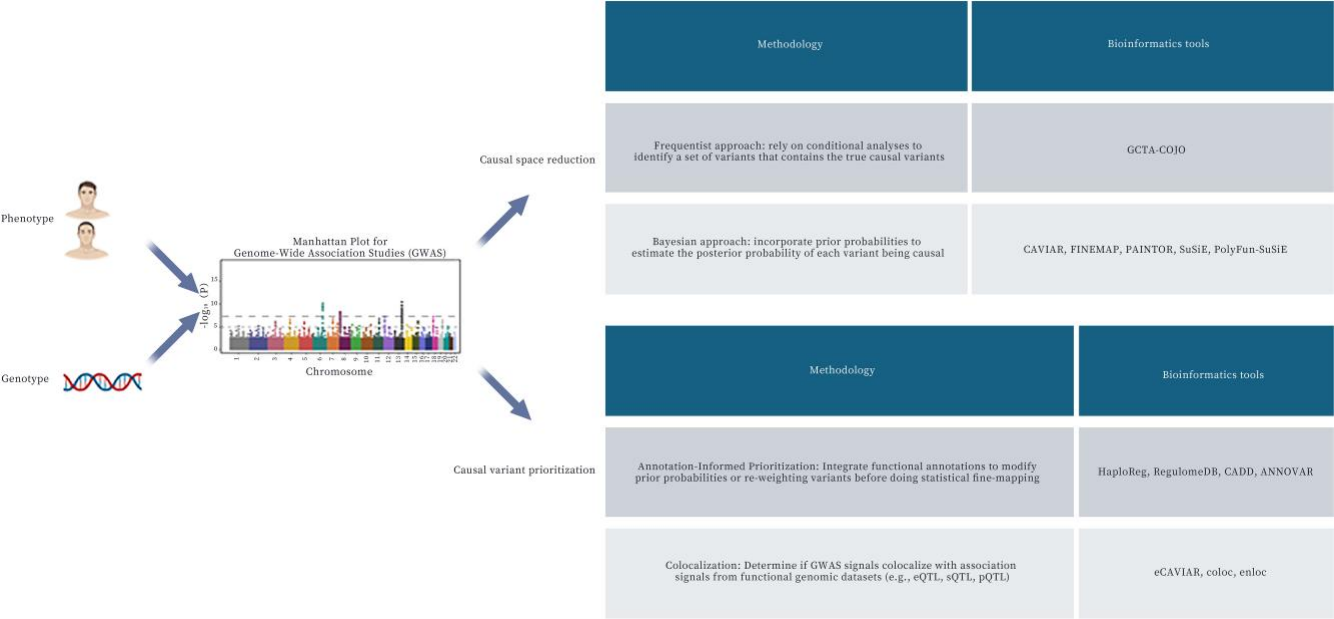
Overview of traditional statistical genetics methods for mapping putative causal variants (created with BioRender.com).

Post-GWAS analyses for causal inference can be broadly classified into two categories: causal space reduction and causal variant prioritization (Fig. 1). Causal space reduction aims to narrow down the search space for potential causal variants to mitigate the confounding caused by LD. This step includes frequentist and Bayesian methodologies, where the former aims to identify independent signals within a locus and the latter quantify the probability that a given SNP is causal. Frequentist-based approaches, including conditional and joint analysis (GCTA-COJO),^25^ employ stepwise regression models that iteratively adjust for the effects of lead SNPs to identify independent association signals. Bayesian fine-mapping approaches estimate the posterior probability of causality for genetic variants by modelling the joint distribution of effect sizes across a locus. Early pioneering methods, such as CAVIAR^26^ and PAINTOR,^27^ establish this core framework. While both account for LD, PAINTOR is designed to integrate functional genomic annotations to inform prior probabilities of causality. Subsequent methods like FINEMAP^28^ dramatically improve computational efficiency for this task, while SuSiE^29^ introduce a novel iterative approach to decompose regional signals into a sum of single effects, yielding highly interpretable credible sets. Sophisticated functional integration methods like PolyFun + SuSiE^30^ has also been developed, where SNP-specific functional priors are first estimated and then be used within the SuSiE framework to perform fine-mapping. These methods generate credible sets, which represent the minimal subset of SNPs that collectively capture a high probability of containing the true causal variant. The effectiveness of these methods is contingent upon high-quality GWAS summary statistics and accurate LD reference panels, as misestimation of LD structure can lead to inflated credible sets or erroneous prioritization of variants.

After obtaining a list of independent putative causal variants, additional functional characterizations that integrate omics data can be applied to build a biological narrative around its potential role in disease. The central principle is that causal variants are more likely to reside in functionally active regions of the genome that regulate the expression and function of target genes. This is put into practice by systematically layering multiple streams of evidence. A primary layer comes from connecting variants to gene expression, using large-scale eQTL resources like GTEx to see if a variant influences overall transcript abundance, or splicing QTL (sQTL) maps to see if it alters isoform usage.

To systematically collate this disparate information for any given variant, various variant annotation bioinformatics tools have been developed. Annotation portals like HaploReg^31^ and RegulomeDB^32^ serve as central hubs, integrating vast amounts of data from consortia like ENCODE^33^ and Roadmap Epigenomics.^34^ With these tools, a researcher can instantly see if a variant overlaps with key regulatory features, such as open chromatin regions identified by ATAC-seq, enhancer- or promoter-associated histone marks like H3K27ac, or known transcription factor binding sites. Command-line tools like ANNOVAR^35^ perform this annotation on a massive scale, efficiently mapping millions of variants to their corresponding gene features. Complementing these databases are sophisticated scoring algorithms that distill this complex information into a single, quantitative metric of potential impact. For example, CADD (Combined Annotation Dependent Depletion),^36^ provides a widely used score that predicts the deleteriousness of a variant by integrating dozens of annotations, from evolutionary conservation to functional genomic data. By leveraging this ecosystem of tools, researchers can prioritize a list of associated variants into a set of biological-relevant candidates.

Beyond annotation-informed prioritization, colocalization analysis provides a critical statistical framework for disentangling the causal relationships between genetic variation and molecular traits. Unlike approaches that merely assess correlations, colocalization statistically tests the probability that a GWAS signal and a molecular trait (eg, an eQTL) share a common causal variant, thereby reducing false positives due to LD. Early foundational methods, such as coloc^37^ and eCAVIAR,^26^ establish this framework using GWAS summary statistics. coloc, a widely used Bayesian method, evaluates competing hypotheses to determine if a shared causal variant exists, but is limited by its core assumption of a single causal variant per locus. While eCAVIAR extended the model to allow for multiple causal variants, its practical application can be limited in regions with complex signals. Recognizing these limitations, recent studies have increasingly shifted towards more robust methods that integrate multi-variant fine-mapping directly into the colocalization pipeline. This modern approach first identifies credible sets of potential causal variants for each trait independently before evaluating the overlap. A leading example of this new paradigm is coloc-SuSiE. This method leverages SuSiE to fine map both the GWAS and the molecular QTL signals, allowing for multiple independent causal variants for each. It then performs colocalization analysis on the fine-mapped signals for each pair of credible sets, providing a granular view capable of distinguishing scenarios where two loci partially colocalize. Methods like enloc^38^ also leverage fine-mapping results but focus on jointly estimating the genome-wide enrichment of colocalized QTL–GWAS signals and computing well-calibrated regional colocalization probabilities to identify high-confidence loci.

To gain insights into potential causal mechanisms beyond the variant level, MR is often performed to test whether changes in molecular traits (eg, gene expression, splicing, epigenetic marks) cause changes in disease risk. It's built on the principle that random segregations of alleles during inheritance mimics the random assignment in a clinical trial. In this framework, the genetic variants serve as instrumental variables for the exposure. Because these genetic instruments are assigned at birth, they are largely independent of confounding lifestyle or environmental factors and cannot be altered by the onset of disease, thus providing robust protection against confounding and reverse causality. Traditionally, MR required genetic data, exposure data, and outcome data from a single group of individuals. This was often difficult to obtain. Two-Sample MR revolutionized the field by allowing researchers to perform MR using just the summary statistics from two separate GWAS.^39^ This is powerful because it allows researchers to harness the statistical power of massive, publicly available GWAS datasets. While MR is powerful, rigourously testing for pleiotropy, where a genetic instrument affects the outcome via a pathway independent of the exposure, is an essential step. The TwoSampleMR toolkit facilitates this by including several key sensitivity analyses. Methods like MR-Egger regression are used to detect and adjust for directional pleiotropy, while Weighted Median MR offers a robust estimate of the causal effect, provided that at least 50% of the instruments are valid. In contrast, GSMR is a distinct method that test for a causal association using multiple genetic variants, while accounting for the LD between them.^40^ Rather than treating pleiotropy testing as a separate, post hoc analysis, GSMR incorporates a pleiotropy detection step directly into its algorithm by actively filtering out outlier instruments that show heterogeneity before providing a causal estimate.

Despite its strengths, MR remains dependent on several key assumptions, including the correct specification of instrumental variables, absence of confounders, and exclusion of reverse causality. Violations of these assumptions can lead to misleading causal inferences, particularly in complex traits where multiple regulatory mechanisms interact. Additionally, the quality of the underlying GWAS and functional QTL datasets critically influences MR reliability. If the instrumental variables are weakly associated with the exposure or if exposure-outcome relationships are confounded by hidden genetic correlations, MR estimates may be biased or lack statistical power.

These well-established statistical genetics approaches have demonstrated remarkable success in uncovering genetic mechanisms underlying complex diseases. For example, A study integrating brain-derived eQTLs with MR and colocalization successfully identified five genes—ACE, GPNMB, KCNQ5, RERE, and SUOX—as promising therapeutic targets for Alzheimer’s disease, Parkinson’s disease, multiple sclerosis, and amyotrophic lateral sclerosis.^41^ Notably, GPNMB, a gene encoding the transmembrane protein glycoprotein nonmetastatic melanoma protein B, was further studied experimentally to elucidate its molecular role in PD and prioritized as a therapeutic target for PD.^42^

### 1.2. Why are traditional statistical genetics methods alone insufficient?

Current GWAS is dominated by linear additive models, which fail to capture the complex, nonlinear relationships underlying disease aetiology. Despite the discovery of thousands of disease-associated loci through traditional linear model-based GWASs, these variants explain only a fraction of genetic heritability for complex traits.^43^ This missing-heritability gap is largely due to the large number of variants whose effect sizes too small to reach genome-wide significance,^25,44,45^ which is an issue that can be addressed primarily by increasing cohort sizes rather than by computational advances.^46^ In contrast, other contributors to the heritability gap, such as phenotypic heterogeneity, gene–environment interactions, and nonadditive mechanisms, can benefit more from computational advancement.^47,48^ Specifically, evidence suggests that genetic epistasis (ie the interactions between genetic variants) significantly enhances risk prediction and provides deeper mechanistic insights into disease pathology. For instance, a study of 46,000 breast cancer cases and 42,000 controls found that SNP-SNP interactions accounted for additional risk variance undetected in single-locus tests.^49^ In primary open-angle glaucoma, incorporating SNP interactions increased the explained heritability from 2.1% to 3.5%, demonstrating the importance of modelling epistasis.^50^ A study on Parkinson’s disease found that risk variants in the HLA region do not act independently; rather, their effects are modified by the presence of other SNPs.^51^ Conditional analyses revealed that multiple independent SNPs (rs3129882, rs2844505, rs9268515) interact to influence disease susceptibility, with certain haplotypes significantly increasing risk compared to individual variants alone. This suggests that GWAS focusing only on single-SNP associations may misidentify true causal loci by failing to account for epistatic effects.

Many approaches attempt to address these limitations by analyzing epistasis at the gene or protein level, but they often rely on marginal SNP associations and miss true SNP-SNP interactions. Gene-level aggregation assumes that interactions manifest at the functional protein level, which holds for some pathways but not universally. For example, SNPs in noncoding regions, which regulate expression via transcriptional enhancers and chromatin accessibility, exhibit strong combinatorial effects that cannot be inferred from protein-protein interaction networks alone.^52^ Ignoring SNP-level epistasis risks missing key mechanistic insights, particularly in complex polygenic diseases where transcriptional regulation plays a major role.

Although various statistical- and data-mining-based methods to detect epistasis from population genetics studies have been developed over the past two decades, these approaches have increasingly struggled to keep pace with the complexity of modern datasets. Early regression-based models, while foundational, became computationally impractical due to the combinatorial explosion of SNP interactions. Methods like the Kirkwood Superposition Approximation improved efficiency but remained limited to pairwise interactions and suffered from false positives in low minor allele frequency scenarios.^53^ Bayesian approaches, such as Bayesian Epistasis Association Mapping, incorporated prior knowledge and probabilistic frameworks but were computationally intensive and reliant on accurate priors.^54^ Nonparametric strategies like Multifactor Dimensionality Reduction captured higher-order interactions but were computationally prohibitive and sensitive to imbalanced datasets.^55^ SNP-set models, including Logistic Kernel Machines (LKM)^56^ and weighted Principal Component Analysis (wPCA),^57^ provided greater flexibility relative to single-SNP tests but depended on predefined SNP groupings, limiting their adaptability to unknown epistatic architectures. Consequently, despite these advances, traditional approaches still falter when confronted with higher-order epistasis, sparse genotype combinations, and the computational burden imposed by exhaustive searches or rigid statistical assumptions.

Another critical limitation of traditional statistical GWAS and post-GWAS approaches is their inability to effectively integrate multi-omics data to prioritize causal genetic elements for complex diseases. Multi-omics integration is critical in distinguishing true causal effects from statistical correlations by integrating functional annotations and regulatory elements. However, while statistical methods can isolate associations between specific variants and molecular phenotypes, they struggle to model the complex regulatory relationships spanning multiple omics layers. This limitation is particularly relevant in complex polygenic diseases, where genetic risk variants often reside in noncoding regulatory regions and exert their effects through transcriptional and post-translational mechanisms. In Alzheimer’s disease, for example, GWAS-identified risk loci such as *APOE*, *BIN1*, and *CLU* affect chromatin accessibility and transcription factor binding in microglia, ultimately influencing tau aggregation and amyloid-beta clearance.^58^ However, a standard eQTL-based approach may identify a SNP influencing *BIN1* expression but fail to capture downstream proteomic interactions, such as tau phosphorylation, that ultimately drive disease progression. Similarly, in Parkinson’s disease, risk variants in *SNCA* (α-synuclein) and *LRRK2* contribute to disease risk, but their pathogenic impact is mediated by post-translational modifications such as phosphorylation and ubiquitination, which influence α-synuclein aggregation and mitochondrial dysfunction.^59^ An optimal framework for identifying true molecular drivers of complex diseases must integrate genetic, epigenetic, transcriptomic, and proteomic data, allowing for the detection of both individual and interaction genetic elements that causally influence disease susceptibility.

## 2. Emerging deep learning approaches for causal learning with population genetic and multi-omics data

### 2.1. Deep learning enables efficient high-order epistasis detection

**Fig. 2.**
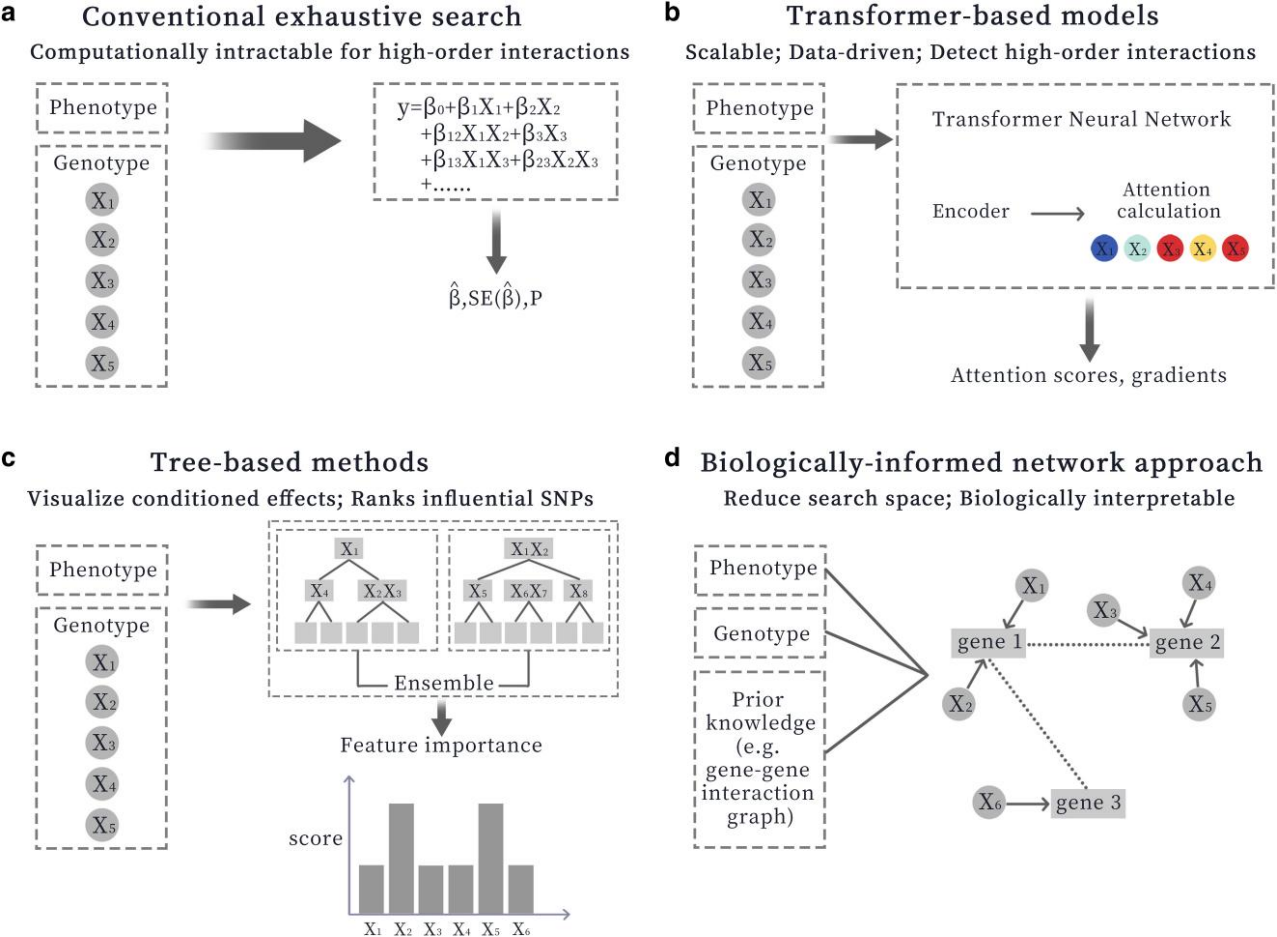
Schematics of variant epistasis detection approaches.

As the hypothesis generation step for causal genomics, the ability to comprehensively discover disease-associated variants and variant interactions is essential. Compared to standard linear model-based GWAS that struggle to detect high-order SNP-SNP interactions, deep learning methods are being developed as powerful tools for genome-wide discoveries (Table 1). A key aspect enabling their application is the initial transformation of complex biological data into structured numerical inputs that these models can interpret. For instance, genomic data such as SNPs are commonly converted using genotype encoding, one-hot encoding, or by breaking down sequences into k-mers. Architectures like transformers often incorporate position embeddings to maintain the order of elements. This capability to scale to and extract features from genomic data is crucial for identifying intricate genetic interactions involved in disease development. A significant advancement in epistasis detection involves using transformer-based structures, which have proven effective in identifying SNP-SNP relationships that go beyond conventional second-order analyses (Fig. 2). Unlike conventional exhaustive techniques that suffer from computational limitations due to the exponential increase in possible SNP combinations, transformers use attention mechanisms to selectively detect high-order relationships and weight the importance of different genomic loci. For example, Graça et al. use attention scores and gradient-based relevance measures to maintain explainability while allowing efficient scalability to large GWAS datasets.^60^ Interestingly, they show that their models can identify interactions up to the eighth order, an achievement that was virtually impossible with conventional statistical techniques.

**Table 1.** Comparative overview of statistical versus deep learning approaches in causal genomics.

Relevance to causal genomics	Methodology		Strengths	Limitations
**Hypothesis generation:** **Genome-wide discovery of potentially causal genetic variants**	Traditional statistical methods	Linear effect models	Simple to execute; high interpretability	Additive-only effects
		Data-mining-based methods	Explicit interaction testing, allowing for exhaustive search in principle	Combinatorial explosion; multiple testing burden
	Emerging deep learning approaches	Transformer-based methods	Able to capture long-range, higher-order interactions	Requires larger sample sizes to avoid overfitting
		Random forest-based variable importance analysis	Relatively robust to overfitting; generate importance ranking of SNPs	LD-induced bias
		Network-guided search with biological priors	Reduce search space; enhanced interpretability	Dependence on prior quality; over-reliance on existing biology may miss novel loci
**Hypothesis refinement:** **Analytical functional characterization with omics data**	Traditional statistical methods	Colocalization analysis	Explicitly test whether a disease association and a molecular trait share a common causal variant; LD robust	Analyse omics data as separate, individual layers; struggles to explain complex regulatory relationships that span multiple omics layers
		Mendelian randomization	Built on well-established principles of causal inference; provide quantifiable measures of confidence	
	Emerging deep learning approaches	Multi-modal integration strategies	Identify cross-modal dependencies	Prone to overfitting with small sample sizes; lack standardized framework for statistical rigour

Another promising approach for flagging SNP interactions is random forest-based variable importance analysis. Random forest models have been applied to capture epistatic SNP effects by leveraging their intrinsic variable importance measures. These ensemble tree methods can model nonadditive interactions without prespecifying SNP pairs, as splits on one SNP can condition on another, revealing complex joint effects. Approaches like the recurrent relative variable importance measure (r2VIM) build on this by running random forests multiple times to identify consistently important SNPs involved in interactions.^61^ A key strength is that random forests are relatively robust to overfitting and can naturally sift through high-dimensional SNP data to highlight candidates. However, their importance scores can be biased by LD and other correlations. For example, if a truly causal SNP is in high LD with a noncausal variant, the proxy variant may also receive a high importance score, making it hard to distinguish the true interacting loci. Thus, while random forest variable importance aids in detecting epistasis by ranking influential SNPs, additional steps are often required to disentangle genuine interaction effects from spurious importance due to LD or main effects.

To tackle the challenge of interpreting epistasis, network-based strategies that integrate prior biological knowledge into the detection process is advantageous. These methods map SNPs to genes or functional elements and constrain searches to biologically plausible interactions using known networks of gene–gene or protein–protein relationships. For instance, Hoffmann *et al.* (2024) construct a “SNP–SNP interaction network” by linking SNPs whose corresponding genes physically interact or participate in the same protein complex. Their algorithm (NeEDL) then searches for clusters of SNPs in this network that jointly associate with disease, seeding the search in regions of the network to find high-scoring epistatic sets. Similarly, Wang *et al.* (2022) introduce EpiHNet,^62^ which fuses statistical evidence with multi-layer biological networks. It combines an SNP co-association network from case-control data with a heterogeneous network of SNP–miRNA–lncRNA interactions, producing a composite graph in which modularity-based clustering is used to discover groups of interacting SNPs. By incorporating gene regulatory and protein interaction priors, these network-driven methods dramatically reduce the search space to focus on SNP combinations that are biologically coherent, thereby improving power and result interpretability. Indeed, EpiHNet was shown to more precisely detect high-order SNP interactions than competitive non-network baselines, thanks to its clustering of SNPs into functionally related modules and the exclusion of implausible pairs. The flip side of these network-based methods include a dependence on known interaction data, as truly novel epistatic mechanisms involving genes with no prior network connection might be overlooked due to the algorithm’s prior filtering. In addition, the complexity of building these SNP networks and the need for curated databases like dbSNP and protein interaction maps means the approach must balance thoroughness of search with the risk of missing biology that falls outside established networks.

Overall, these methods demonstrate the growing potential of deep learning to reveal nonadditive genetic effects that drive complex diseases, providing an efficient and comprehensive way to discover potential causal genetic factors for further prioritization. The next section explores the advantages of deep learning approaches in refining the hypothesis on the identified genetic factors with multi-omics data.

### 2.2. Deep learning approaches for integrating multi-omics data

**Fig. 3.**
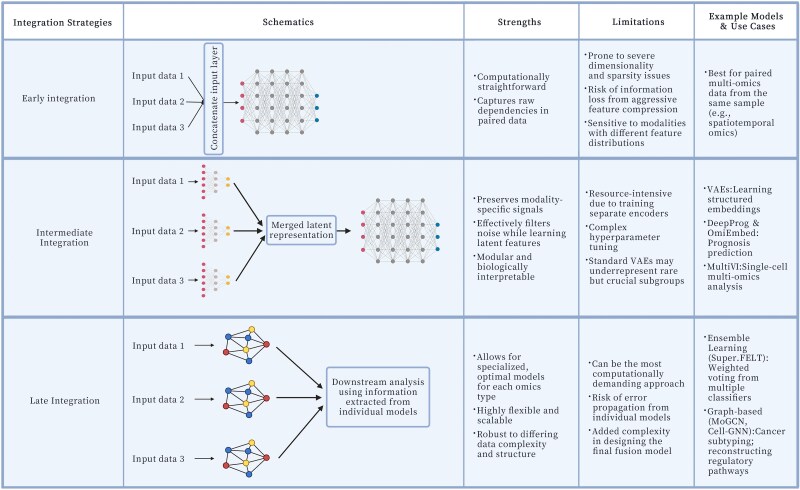
Deep learning-based multi-omics integration paradigms (created in BioRender.com).

Deep learning also offers a unique avenue for multi-omics integration that moves beyond the traditional post-GWAS methods that use each omics layer separately for functional annotation of GWAS results. It enables the discovery of complex interdependencies between genetic, transcriptomic, epigenetic, and proteomic features that are often lost in sequential analyses.^63^ However, multi-omics integration presents significant computational challenges, including differences in feature distributions, modality-specific noise, and the risk of overfitting. To address these challenges, deep learning-based integration strategies fall into three major categories: early, intermediate, and late integration (Fig. 3). Each approach varies in how and when the information from different omics layers is combined, affecting scalability and interpretability.

Early integration concatenates features from all omics layers into a single high-dimensional representation before training a model. This approach is relatively computationally straightforward and is applicable to the newer spatiotemporal omics datasets where paired multi-omics data from the same biological sample are increasingly available. However, caution should be used to address the severe dimensionality and sparsity challenges, particularly when integrating omics modalities with vastly different feature distributions.^64^ Aggressive dimensionality reduction techniques are often necessary to mitigate these issues, but excessive feature compression can lead to the loss of biologically significant signals, thereby limiting the interpretability and predictive power of downstream models.^65^

Intermediate integration offers a more refined approach by learning latent representations for each omics layer independently before combining them in a shared space for downstream tasks. This strategy preserves modality-specific signals while enabling meaningful fusion across data types. Techniques like variational autoencoders (VAEs) have gained prominence in this domain by effectively filtering out noise while preserving biologically relevant features. DeepProg exemplifies this approach, using autoencoders to transform omics data into lower-dimensional embeddings before merging them for prognosis prediction.^66^ Instead of feeding raw multi-omics data directly into a unified model, DeepProg applies unsupervised learning at the feature extraction stage, selecting survival-associated latent variables from each omics-specific autoencoder. This method enhances biological interpretability while minimizing the risk of overfitting. Other frameworks, such as OmiEmbed, employ similar strategies, mapping high-dimensional omics data into structured latent spaces optimized for predictive tasks.^67^ Deep generative models, particularly multi-modal VAEs, have further refined intermediate integration by learning joint probabilistic embeddings that preserve biological structure. For example, MultiVI extends VAEs by explicitly modelling modality-specific noise and batch effects, making them particularly effective for single-cell multi-omics data integration.^68^ However, this modularity introduces critical trade-offs. A fundamental challenge, particularly for precision medicine, is that standard VAEs prioritize dominant signals in heterogeneous populations, potentially under-representing rare but biologically crucial subgroups. This risk of information loss is compounded during feature extraction, where careful tuning is required to prevent the overcompression of important signals in the latent space. Furthermore, this approach is relatively resource-intensive due to the need for training separate encoders and navigating a complex hyperparameter space to ensure reproducible optimization.

Late integration, by contrast, trains separate models on each omics layer before combining their outputs into a final prediction. This approach is especially advantageous in scenarios where different omics modalities exhibit distinct data structures and levels of complexity.^64^ By processing each modality independently, late integration allows for specialized modelling tailored to the statistical properties of each omics type, preventing information loss due to premature fusion. Graph-based methods and ensemble learning techniques frequently facilitate this process by leveraging the strengths of each modality-specific model while capturing cross-modal interactions at a later stage. Deep ensemble learning frameworks mitigates biases associated with any single omics modality by averaging or voting across predictions from distinct models. In Super.FELT, modality-specific encoders first extract feature representations separately from transcriptomic, genomic, and epigenomic data.^69^ These representations are subsequently fed into independent classifiers, and their outputs are aggregated through an attention-weighted fusion mechanism to emphasize the most predictive modalities. This methodology effectively balances model flexibility and integration scalability, making it well-suited for complex multi-omics tasks.

Graph-based methods, such as graph neural networks (GNNs), provide another powerful late integration strategy by structuring omics data into networks where biological entities (eg, genes, proteins, metabolites) serve as nodes and relationships (eg, co-expression, physical interactions, metabolic pathways) are represented as edges.^70^ Unlike conventional machine learning models, which treat omics features as independent variables, GNNs leverage these biological relationships to improve prediction accuracy and interpretability. For instance, MoGCN (Multi-Omics Graph Convolutional Network) refines cancer subtype classification by incorporating pathway-based priors.^71^ MoGCN constructs a heterogeneous graph where nodes correspond to genes and pathways, and edges capture molecular interactions inferred from transcriptomic and epigenomic data. Through iterative message passing, MoGCN learns contextualized embeddings that integrate cross-omics regulatory patterns, improving its ability to distinguish cancer subtypes with high biological fidelity. Similarly, GLUE (Graph-Linked Unified Embedding) applies a graph-based multi-omics integration approach to infer regulatory interactions and unified cell embeddings from unpaired single-cell multi-omics datasets.^72^ Unlike MoGCN, which primarily focuses on bulk-level omics integration, GLUE models cell-type-specific and cross-modal interactions by leveraging a knowledge-based regulatory graph to link omics-specific embeddings. This method effectively captures cell-state transitions and reconstructs lineage differentiation pathways by explicitly modelling relationships between different omics layers, such as linking transcriptional activity to chromatin accessibility in a unified latent space. Despite these advantages, late integration can be the most computationally demanding approach. Training multiple, independent deep learning models requires significant computational resources, and the final fusion model adds another layer of architectural complexity and potential for overfitting. This approach also risks error propagation; if one modality-specific model performs poorly, it can negatively influence the final prediction, potentially obscuring important biological insights even with sophisticated fusion mechanisms like attention.

### 2.3. Deep learning causal discovery methods

Constructing a molecular causal map for a complex disease enables the identification of drug targets with high confidence. While the approaches described above are powerful for identifying statistically significant interactions and annotating them with functional multi-omics data, they primarily uncover complex associations rather than explicit causal relationships. Detecting an epistatic interaction that correlates with changes across multiple omics layers provides a strong biological hypothesis, but it does not inherently reveal the directional, causal pathway. To move from this rich correlational landscape to a predictive causal map of disease, more rigourous deep learning causal discovery frameworks are required. For example, unsupervised causal discovery methods, also known as latent causal representation learning, is particularly advantageous to be applied to integrated datasets to identify hidden causal mechanisms underlying disease etiology.^73^ Methods such as CausalVAE and deep structural causal models (SCMs) have demonstrated their ability to extract meaningful latent representations that capture high-order interactions among molecular elements.^74^ Beyond these approaches, multi-domain causal representation learning presents a novel avenue for integrating heterogeneous datasets that may share latent causal factors. Recent work has provided theoretical guarantees for identifying shared causal graphs across unpaired datasets, addressing a critical limitation in traditional causal inference techniques.^75^ Applying and refining these advanced causal learning techniques to the outputs of epistasis and multi-omics analyses will transform our ability to map disease mechanisms, moving us closer to therapeutic strategies that address root causes rather than symptoms.

We summarized the major deep learning frameworks described above into several structural axes, including the level of supervision, model type, and the nature of the learned representation, to facilitate improved contextualization (Table 2).

**Table 2.** Taxonomy of deep learning frameworks applied to causal genomics.

Deep learning frameworks	Primary task	Supervision level	Model type	Representation	Key genomic challenge addressed
**Transformer-based models**	Epistasis detection	Supervised	Discriminative	Implicit (embeddings)	High-dimensionality, complex nonadditive interactions
**Tree-based models**	Epistasis detection	Supervised	Discriminative	Implicit (feature importance)	High-dimensionality, noisy features
**Network-based models**	Epistasis detection	Supervised/Semi-supervised	Discriminative	Explicit (biological graph)	Reducing search space, integrating biological priors
**Variational autoencoders**	Multi-omics integration	Unsupervised	Generative	Implicit (latent embeddings)	Data heterogeneity, noise reduction, dimensionality
**Graph neural networks**	Multi-omics integration	Supervised/Semi-supervised	Discriminative	Explicit (interaction graph)	Data heterogeneity, modelling known relationships
**Latent causal representation learning models**	Causal structure discovery	Unsupervised	Generative	Implicit (latent causal graph)	Discovering hidden causal factors from observational data

## 3. Challenges of deep learning models in genomics research

Current deep learning models demonstrate excellent capabilities for recognizing complex non-linear hierarchical patterns in multi-omics and genetic datasets but remain in their initial development phase while facing multiple challenges.^76,77^ The main ones include overfitting and the lack of interpretability or statistical rigour.^78^ The learning patterns that a deep learning model detects from training data can sometimes produce noise instead of generalizable relationships which leads to poor performance when testing new data.^79^ Multi-omics data increases this problem by introducing various feature spaces that bring different types of noise and batch effects. When combining transcriptomics, epigenomics, and proteomics data for analysis they produce modality-specific noise that deep learning models may confuse with relevant patterns.^80,81^ The generation of false associations together with missed subtle biologically relevant patterns through overfitting, resulting in unreliable findings that slow down our understanding of disease mechanisms.^79,82^

In preventing overfitting, while standard regularization techniques like dropout and L2 penalties provide a first line of defence, they are often insufficient to address the structured noise inherent in multi-omics data and can indiscriminately penalize biologically relevant signals from rare variants or small patient subgroups.^76^ True generalizability requires imposing a stronger and more appropriate inductive bias, guiding models to learn representations that are invariant to known confounders. One strategy involves adversarial learning, where the model is explicitly trained to be invariant to undesirable factors. For example, by incorporating a counterfactual learning objective, models like CRADLE-VAE can learn to disentangle true biological variation from technical batch artefacts by forcing its representations to be uninformative of the batch origin.^83^ This approach moves beyond simple data normalization to actively regularize the model’s latent space, ensuring that learned features are robust to technical noise. A recent transformative shift for combating overfitting comes from the paradigm of self-supervised and pretrained foundation models. By pretraining on massive, unlabelled genomic datasets, such as entire reference genomes, models like GENA-LM learn the fundamental syntax and grammar of DNA sequences.^84^ This pretraining step imbues the model with a powerful, generalizable understanding of genomic principles. When subsequently fine-tuned on a smaller, task-specific dataset, the model is far less susceptible to overfitting because its learning is constrained by the robust, pretrained feature space. Despite these advances, preventing overfitting is a persistent issue in applying deep learning in genomics research and needs continuous innovation to develop better generalizable techniques.

Another fundamental limitation of deep learning methods in genomics is the absence of a standardized statistical framework. The field of drug target discovery places greater importance on both interpretable deep learning models and statistically sound results rather than just predictive model performance. Traditional statistical analysis establishes robust hypothesis testing frameworks which generate *P*-values and confidence intervals to measure statistical significance and uncertainty. The optimization of deep learning models for predictive accuracy produces results without inherent statistical metrics and standardization which creates difficulties for studying different studies. Model architecture differences combined with variations in hyperparameter tuning and data preprocessing pipelines lead to inconsistent results that fail to reproduce. As noted by Kang et al., deep learning model configuration variations in multiple studies about multi-omics data integration produce results that are challenging to reproduce and interpret.^76^

With the distinct characteristics of traditional causal inference and deep learning causal learning, we believe that the true potential of deep learning in causal genomics lies not in replacing traditional causal inference frameworks, but in augmenting them. Hybrid methods that aim to merge the modelling power of deep learning methods with the interpretability of traditional statistical techniques will enable researchers to tackle complex genomic data while moving towards understanding causal molecular mechanisms in complex diseases.

## 4. Future directions: integrating deep learning with statistical principles

Advancing causal discovery in complex diseases requires novel methodologies that unite the complementary strengths of deep learning and traditional statistical genetics. A crucial example is to fuse the interpretability of statistical models with the predictive power of deep learning. Bayesian neural networks offer a powerful path forward by treating network weights as probability distributions rather than single point estimates. This allows for principled uncertainty quantification. The NN-Bayes framework is an excellent example, designed specifically for genomic prediction and GWAS.^85^ It integrates the well-established “Bayesian Alphabet” models into the network's first layer. This hybrid design preserves the genomic interpretability of traditional Bayesian regression, such as allowing the calculation of posterior inclusion probabilities (PIPs) for individual SNPs, while leveraging the nonlinear modelling capabilities of the subsequent neural network layers to capture complex genetic effects like epistasis. Similarly, pretrained sequence-to-function models like Enformer^86^ and DeepSEA,^87^ which predict tissue-specific expression or chromatin effects from DNA sequence alone, can be integrated directly into statistical pipelines. Their functional importance scores are now routinely used as prior weights in Bayesian fine-mapping tools, shrinking credible sets and helping to pinpoint causal variants in GWAS loci.

Beyond enhancing existing models, this synthesis is creating new avenues for causal inference. One such promising hybrid is Deep Mendelian Randomization (DeepMR).^88^ Traditional MR uses genetic variants, typically identified from linear GWAS models, as instrumental variables to infer causality. The DeepMR approach improves upon this by using a deep learning model to learn a powerful, nonlinear polygenic score from raw genotype data to serve as a more comprehensive and robust instrumental variable. By better capturing epistatic and nonadditive effects, such an instrument can provide more power and precision in the MR framework.

This integration is also transforming how we model causal networks and disentangle biological mechanisms. Deep SCMs that leverage normalizing flows can now perform counterfactual sampling.^89,90^ When applied to eQTL data, these models can infer directed causal graphs that supply causal priors for fine-mapping or TWAS pipelines, replacing simple correlation with directed causal hypotheses. Other methods tackle the challenge of confounding in high-dimensional data. For instance, CRADLE-VAE^83^ uses a counterfactual swap mechanism to disentangle batch artefacts from true biological signal in single-cell RNA-seq data, while CoCoA-diff^91^ frames differential expression as a potential-outcome problem, using a VAE to learn and adjust for latent confounders. These approaches inject the flexibility of deep learning while retaining the rigourous statistical logic of classical methods, leading to more trustworthy results.

The future of complex polygenic disease research lies in the development of integrative frameworks that synergize deep learning’s ability to model complex, high-dimensional data with the statistical rigour of causal inference. Such a framework will not only deepen our understanding of disease etiology but also strengthen the foundation for experimental validation. As methodological advances continue to bridge the gap between data-driven discovery and causal interpretation, the field will be better positioned to identify disease-driving mechanisms and ultimately develop therapies that alter, rather than merely alleviate, the course of these devastating conditions.
